# Supplementary material for: Remote homology and functional genetics unmask deeply preserved Scm3/HJURP orthologs in metazoans
Source: Sci Adv. 2026 Jul 1;12(27):eaeg5356. doi: 10.1126/sciadv.aeg5356 (PMC13322267; doi:10.1126/sciadv.aeg5356)
Supplement: Supplementary file 1 — Figs. S1 to S12 Legends for tables S1 to S4 Legends for data S1 to S3 Legend for movie S1 References [file sciadv.aeg5356_sm.pdf]

Science Advances
AAAS

Supplementary Materials for

Remote homology and functional genetics unmask deeply preserved Scm3/HJURP orthologs in metazoans

Jeremy A. Hollis *et al.*

Corresponding author: Jeremy A. Hollis, jhollis@fredhutch.org; Harmit S. Malik, hsmalik@fredhutch.org

Sci. Adv. **12**, eaeg5356 (2026)
DOI: 10.1126/sciadv.aeg5356

The PDF file includes:

Figs. S1 to S12
Legends for tables S1 to S4
Legends for data S1 to S3
Legend for movie S1
References

Other Supplementary Material for this manuscript includes the following:

Tables S1 to S4
Data S1 to S3
Movie S1

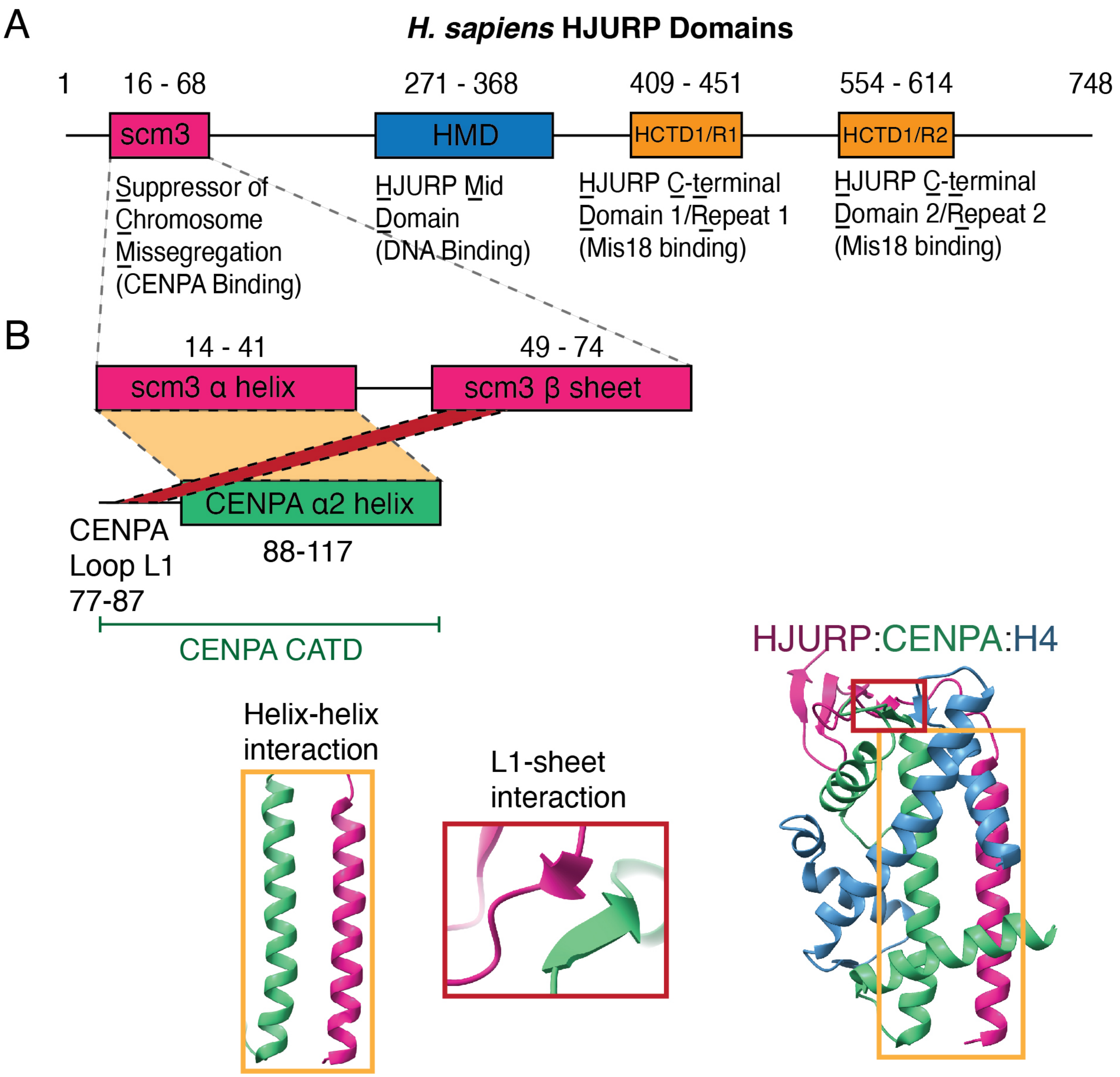

Figure S1. Structure-function relationships of the human HJURP protein. A. A domain schematic for human HJURP. The suppressor of chromosome missegregation (scm3) domain directly binds CENPA/H4 dimers, the HJURP mid domain (HMD) is implicated in DNA binding, and HJURP C-terminal domains (HCTD) 1 and 2 interact with Mis18 to recruit HJURP to the centromere. **B.** A structural overview of human HJURP-CENPA interactions. CENPA forms an antiparallel helix and strand with HJURP. Structure is PDB 3R45[12].

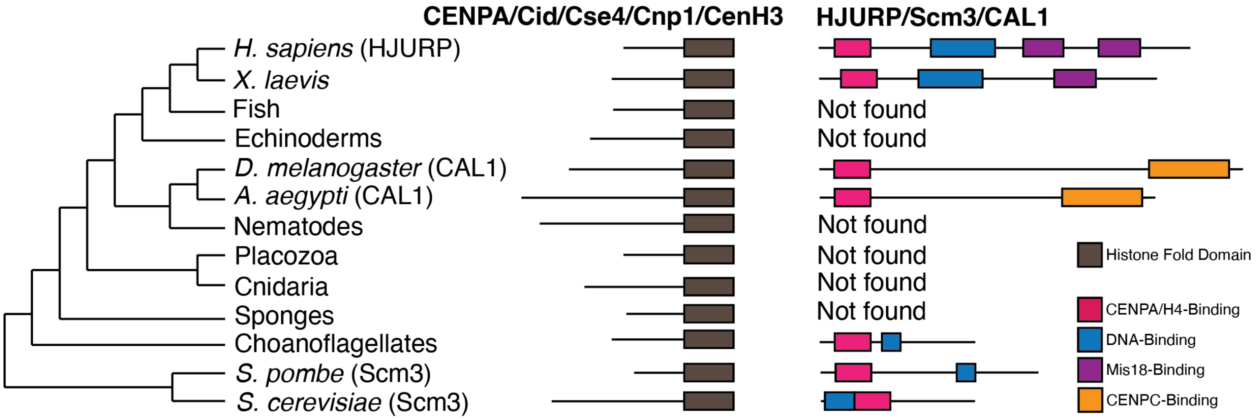

Figure S2. HJURP orthologs had not previously been found in most metazoan clades. A. An unscaled phylogeny showing relationships among major metazoan groups. Select scm3 domain-containing CENPA chaperones have been independently identified and biochemically characterized across the animal tree, yet many species with CENPA appear to lack a scm3 chaperone.

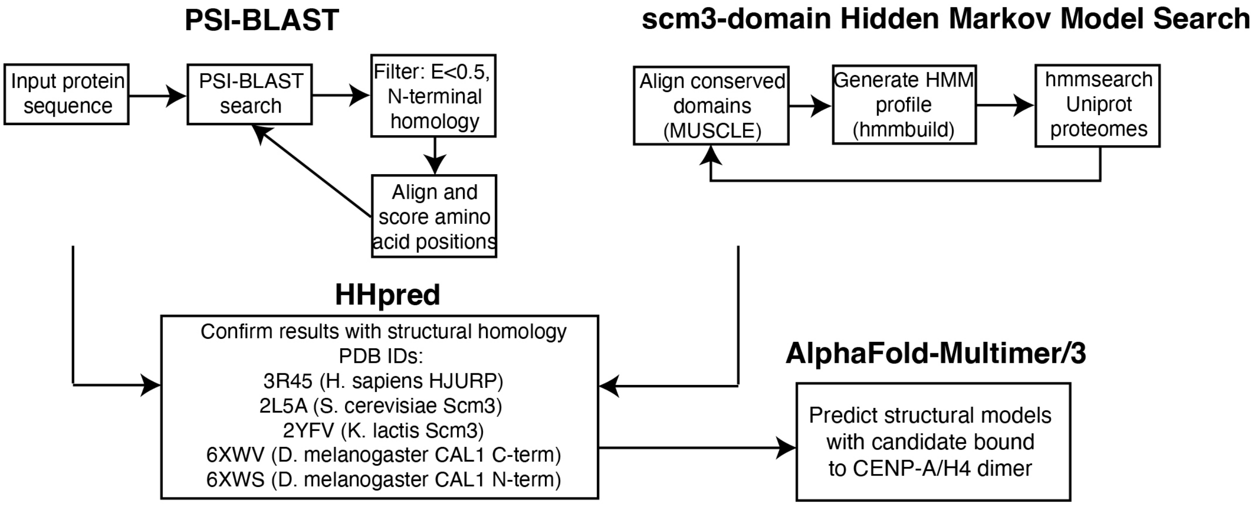

Figure S3. A schematized approach for detecting remote HJURP orthologs. We employed a holistic homology search pipeline integrating sequence and structural data to identify and confirm distant Scm3/HJURP orthologs that previously escaped detection.

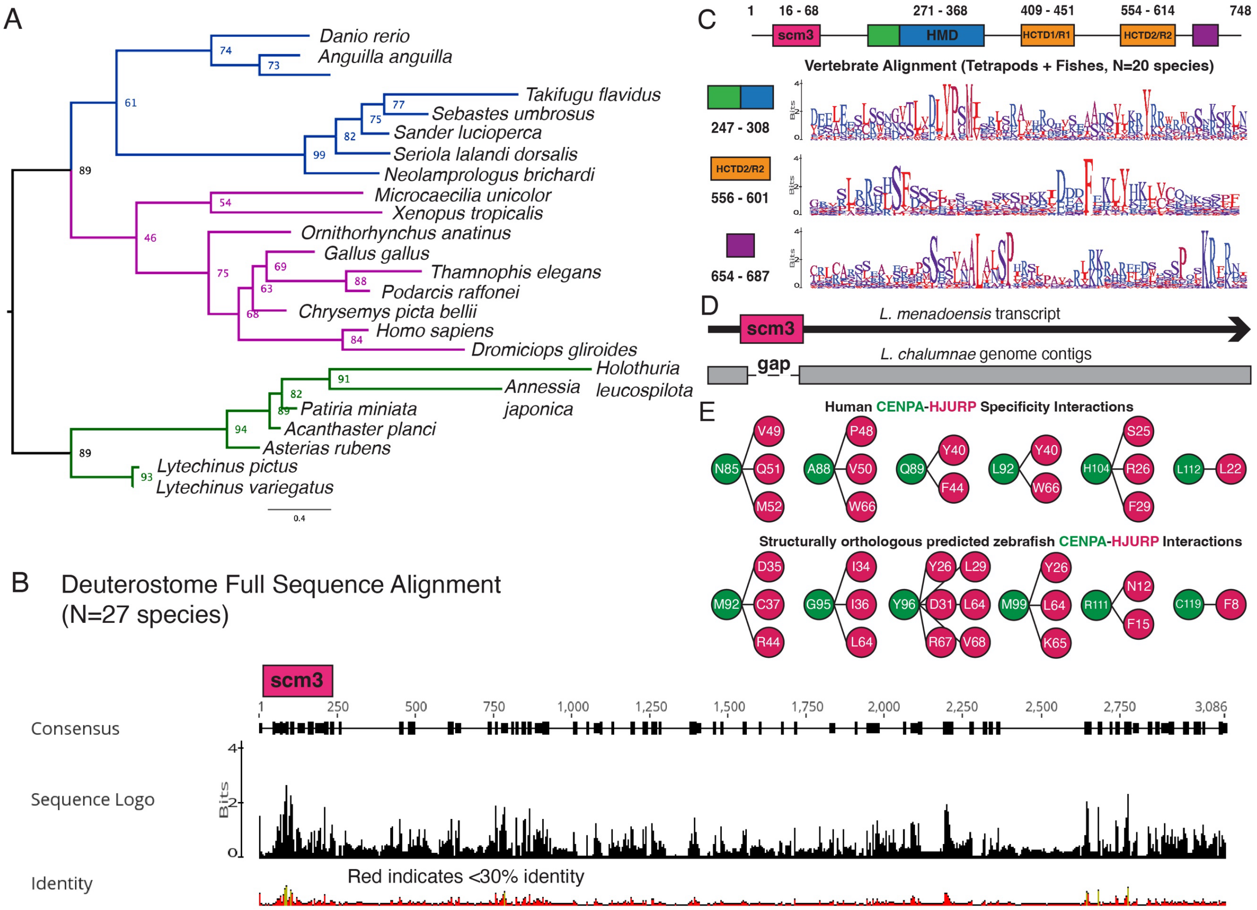

Figure S4. Shared and diverged features of deuterostome HJURPs. A. An expanded, duplicate phylogeny from Fig. 1 of representative deuterostome scm3 domains roughly follows the expected species tree, consistent with a monophyletic origin of HJURP in this clade. Nodes are presented with 1000 ultrafast bootstrap values. **C.** A sequence alignment of full-length putative deuterostome HJURP proteins shows generally poor conservation aside from the N-terminal scm3 domain region. **C.** A domain schematic for human HJURP with additional regions of shared homology between tetrapods and fish displayed as logo plots. Besides the previously identified HMD and HCTD2 domains, we find shared homology N-terminal to the HMD and close to the C terminus. **D.** The *L. chalumnae* genome likely contains a contig gap at the scm3 domain; alignments of translated contig regions to the *L. menadoensis* transcriptome shows coelacanths encode scm3 domain-containing HJURP orthologs. **E.** Structurally orthologous residues that contribute to CENPA-HJURP specificity differ almost entirely between zebrafish and human. Residues are shown that form physical interactions in either the human CENPA/H4/HJURP crystal structure or the predicted zebrafish CENPA/H4/HJURP structure.

A

drHJURP Positively-correlated gene expression	
**Gene**	**r-value**
si:ch211-69g19.2	0.291
tpx2	0.289
cdk1	0.285
plk1	0.283
cenpf	0.282
cdc20	0.28
ube2c	0.28
ccnb1	0.279
mki67	0.279
nusap1	0.278
kpna2	0.277
aspm	0.273
top2a	0.273
kifc1	0.271
mad2l1	0.271
kif11	0.268
aurkb	0.265
aurka	0.263

B

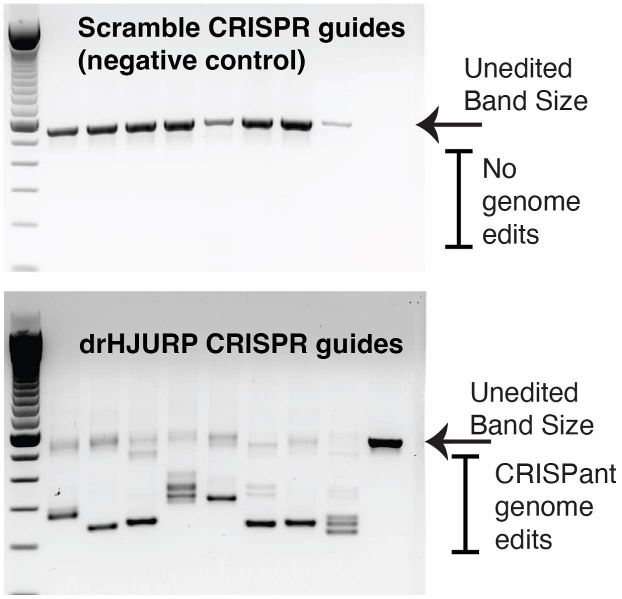

Figure S5. Related to *in vivo* analysis of drHJURP. A. The expression of the putative zebrafish HJURP is most positively correlated with primarily cell cycle genes such as cell cycle kinases (plk1, aurka/aurkb, cdk1) and spindle microtubule regulators (tpx2, aspm, kif11), among others. Data are provided via Daniocell[54]. **B.** Zebrafish CRISPR guides efficiently cause truncation at the HJURP locus (bottom) but non-targeting scrambled control guides fail to do so (top). Bands are PCR products amplified from the HJURP locus of individual CRISPant embryos; farthest right band is amplified from an uninjected control.

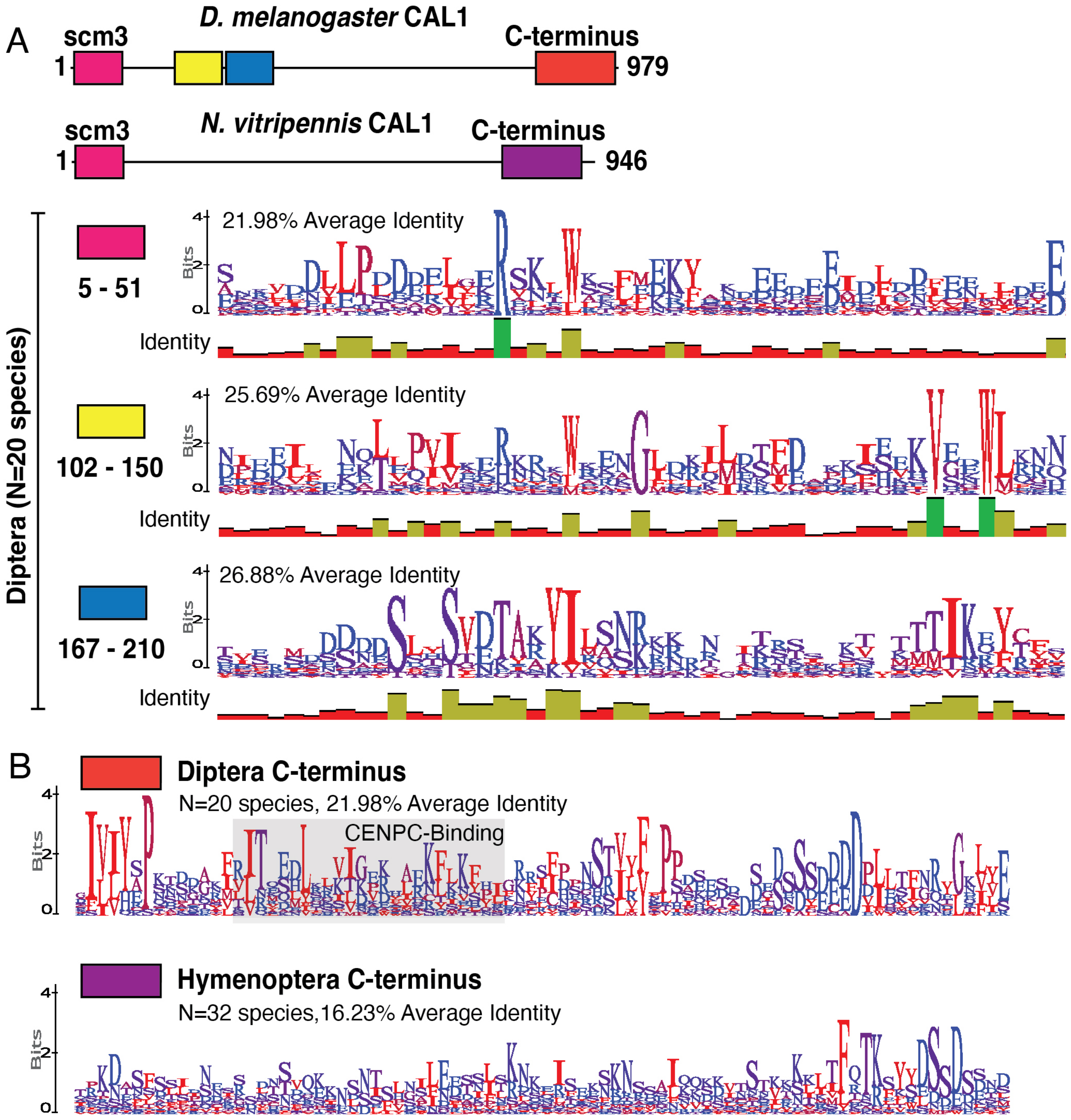

Figure S6. Shared and diverged sequence features of CAL1. A. A domain schematic for *Drosophila* and *Nasonia* CAL1 with additional regions of *Drosophila* homology beyond the scm3 domain displayed as logo plots. **B.** The Hymenopteran CAL1 C-terminus is less constrained than that of Dipteran CAL1.

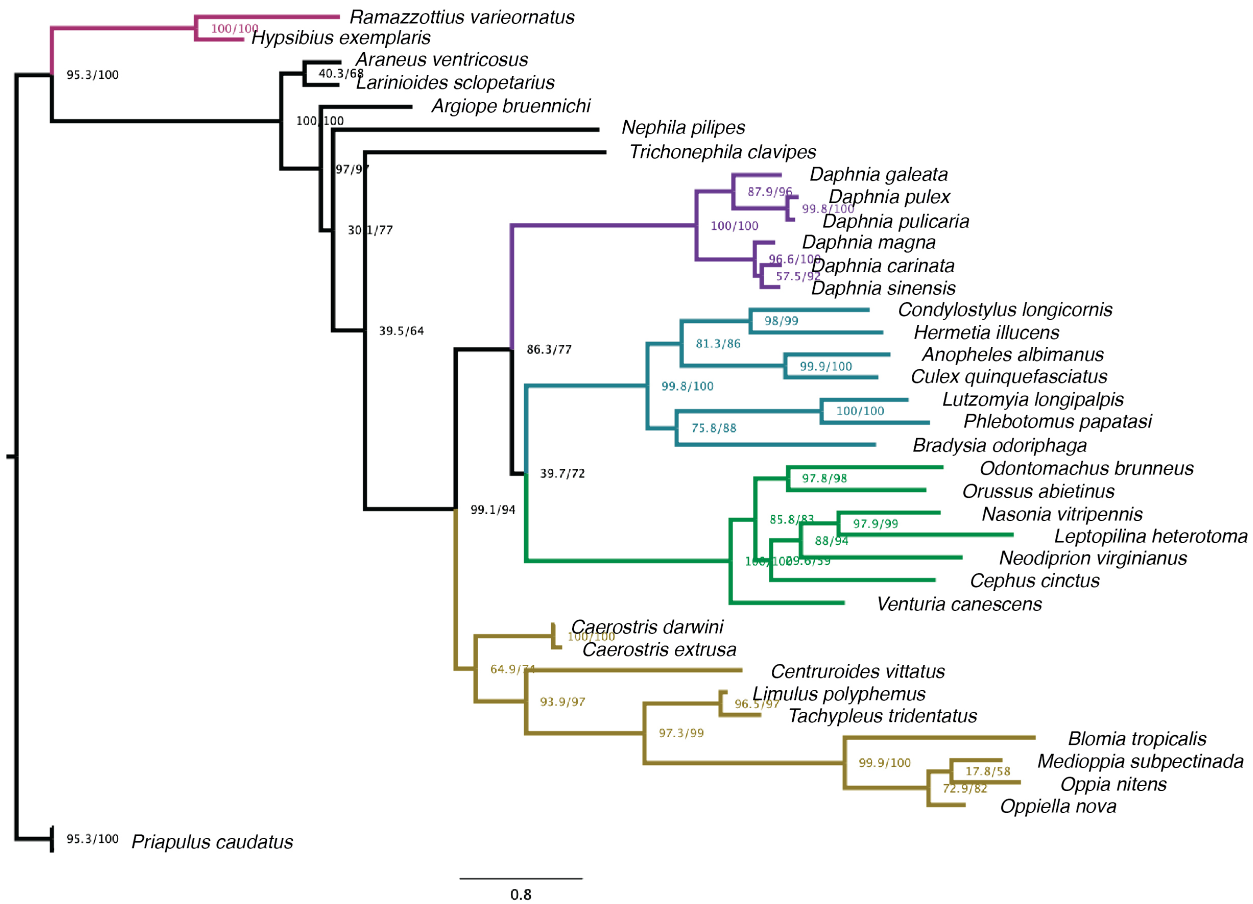

Figure S7. An expanded phylogeny of panarthropod HJURP orthologs. An expanded version of the tree in Fig. 3E generally follows the accepted species tree, although low homology and sparse sequence availability likely contributes to phylogenetic artefacts. Nodes are presented with 1000 ultrafast bootstrap/SH-aLRT replicate values.

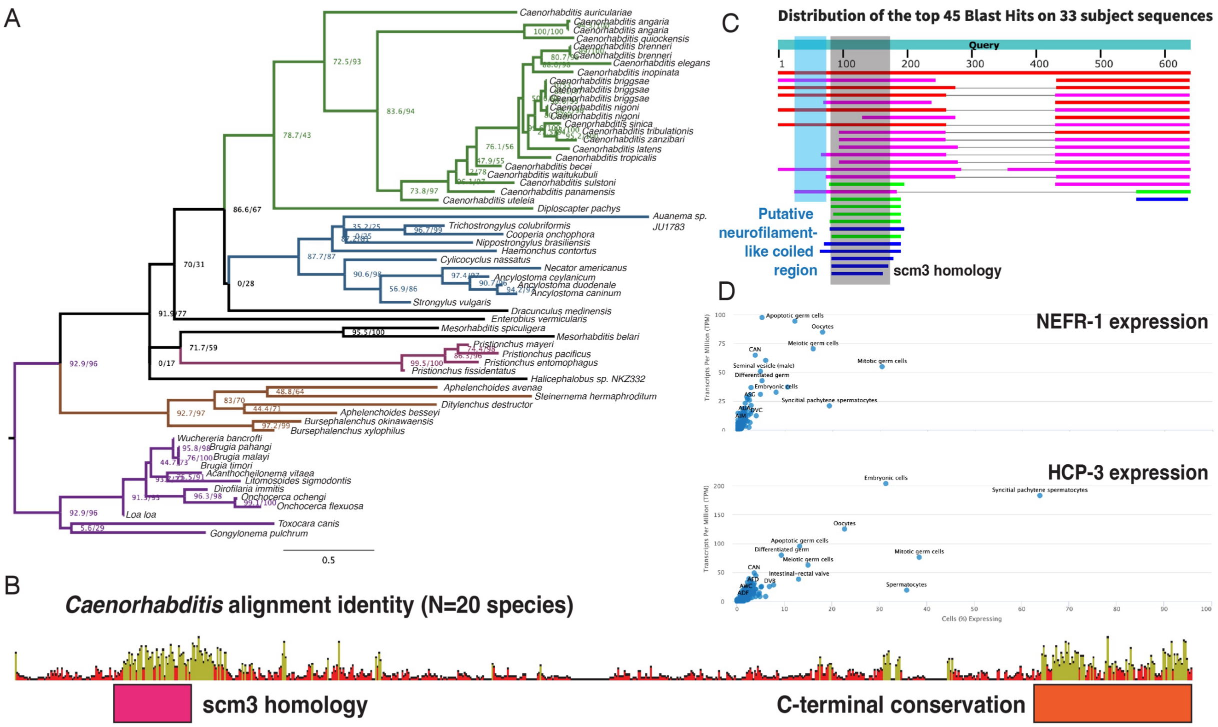

E

GO term enrichment from positively-correlated gene expression (top 100 genes)		
**GO term**	**p-value**	**Representative associated genes**
Cell cycle process	1.28E-31	KCA-1, SGO-1, PLK-1, FZY-1, CDC-25.1, POLE-1, KLP-19, MCM-6, ZIM-1, PLK-3
Nucleus	3.89E-31	POLE-1, GON-4, MRE-11, ALG-5, BRD-1, EXO-1, NASP-2, GLP-1, ICP-1, CTF-4
Nuclear division	1.73E-30	POLE-1, KCA-1, RDE-3, CDT-2, ZYG-1, KLC-1, GLD-3, ORC-1, CYB-2.2, FZY-1
Sexual reproduction	2.43E-30	KCA-1, EGG-2, MARC-3, SPE-39, GLD-3, NOS-1, KLC-1, ZIM-1, RDE-3, PLK-1
Intracellular organelle	5.23E-30	POLE-1, GON-4, MBTR-1, PASH-1, GNA-2, ZTF-1, RIF-1.1, VRK-1, ZTF-8, HIRA-1
Organelle fission	9.50E-30	POLE-1, NOP-1, CTF-4, POLH-1, RDE-3, MCM-6, CYB-2.2, NOP-1, WDR-5.3, ORC-1
Reproductive process	1.37E-29	KCA-1, GON-4, GNA-2, MARC-3, VRK-1, UNC-59, GLP-1, NOS-1, INX-22, ZIM-1, KLP-19
Organelle	1.56E-29	POLE-1, PASH-1, TRUL-1, BRD-1, MUT-7, CITK-2, TAF-8, APTF-3, NPP-22, POLD-1
Cell cycle	2.65E-29	KCA-1, IMA-2, CDT-2, ZYG-1, FZY-1, NOP-1, GLD-3, SZY-4, ICP-1, PLK-1, CDC-25.1

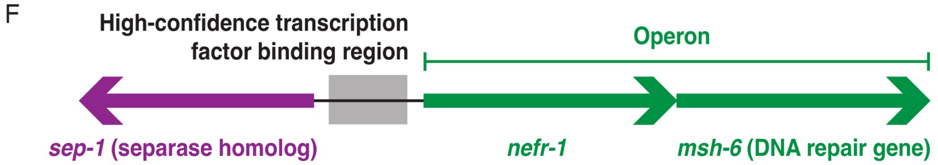

Figure S8. NEFR-1 is a nematode cell cycle protein. A. An expanded nematode phylogeny presented in Fig. 5B roughly follows the species tree, consistent with a monophyletic origin of $HJURP^{NEFR-1}$ in this clade. Nodes are presented with 1000 ultrafast bootstrap/SH-aLRT replicate values. **B.** Conservation pattern of NEFR-1 across *Caenorhabditis* shows there is relatively little sequence conservation outside of the N- and C-terminal regions. **C.** BLAST results show that the scm3 homology and C-terminal regions, rather than the putative neurofilament-like coiled coil region, are the most conserved within nematodes. **D.** NEFR-1, like the worm CENPA homolog HCP-3, is most highly expressed in germline dividing tissue such as oocytes, syncytial spermatocytes, and embryonic cells. Data are from single-cell expression profiles[68]. **E.** NEFR-1 has positive expression correlations with predominantly cell cycle genes. Data on the top 100 genes with positive expression correlation to *nefr-1* and their gene ontology (GO) analyses were provided via the WormBase[101] implementation of SPELL[124]. **F.** Schematic of the *nefr-1* syntenic neighborhood suggests *nefr-1* is expressed with other chromatin-associated cell cycle genes like *sep-1*, required for anaphase chromosome segregation, and *msh-6*, a DNA mismatch repair gene.

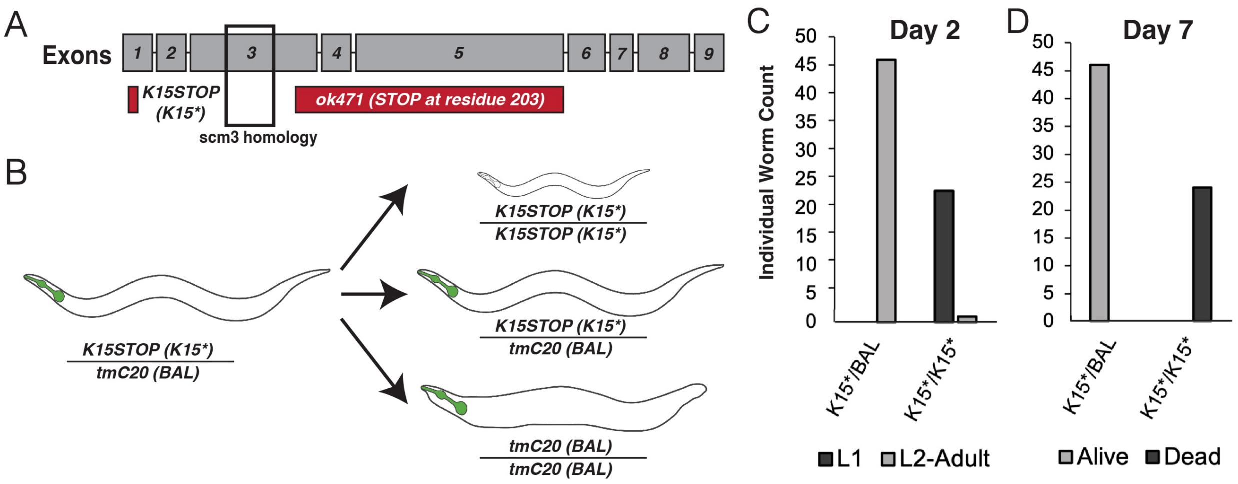

Figure S9. NEFR-1 is an essential gene. A. Gene structure of *nefr-1* shows that the previously characterized truncation allele *ok471* retains the scm3 domain, while the *K15** allele generated in this study, which includes a subsequent frameshift, produces a complete knockout. **B.** Balancer crossing scheme to assess viability of *nefr-1(K15*)* mutants. The *tmC20* balancer confers a GFP pharynx phenotype to heterozygotes and a GFP pharynx + dumpy phenotype to balancer homozygotes. **C.** Worms homozygous for *K15** fail to progress past the L1 stage and **D.** die within a week, while heterozygotes mature and survive into adulthood.

A

GO term enrichment from top 500 mass spectrometry proteins (3xFLAG::NEFR-1)			
GO term	Fold Enrichment	p-value	Representative associated genes
Protein localization to kinetochore	18.48	3.96E-06	npp-10, czw-1, bub-3, rod-1, bub-1
Protein-containing complex localization	9.86	6.07E-04	npp-10, lmn-1, bub-3, bub-1
Mitotic cell cycle process	8.06	2.10E-28	spd-5, him-1, smc-3, mcm-4, tba-2, zyg-9, plk-1, hcp-2
Mitotic DNA replication	24.64	6.66E-07	mcm-4, mcm-6, mcm-2, mcm-1, pola-1
Nuclear cell cycle DNA replication	44.35	2.55E-07	mcm-4, mcm-2, mcm-3, pola-1
Regulation of protein localization to the ER	29.57	1.50E-03	icd-2, icd-1
Negative regulation of protein localization	7.39	1.89E-03	icd-2, icd-1, ftt-2, cdc-48.2
Meiotic cell cycle process	6.95	6.69E-15	klp-18, lin-5, cmd-1, plk-1, scc-3, top-2, hcp-1
Anatomical structure morphogenesis	2.55	6.44E-07	myo-3, unc-70, let-418, unc-54, swsn-1, sma-1, hcp-2
Tube development	4.16	3.18E-04	lam-1, hda-1, let-418, erm-1, pyr-1, spt-16
Spindle assembly	10.44	6.81E-07	paa-1, klp-18, air-1, hcp-2, bmk-1, klp-7, hcp-1
Meiotic spindle organization	19.4	2.71E-08	klp-18, tbb-2, zyg-9, hcp-1, hcp-2
Meiotic chromosome segregation	4.93	8.62E-05	mix-1, plk-1, smc-4, dpy-28, bub-1
Acyl-CoA metabolic process	8.45	3.78E-06	sucg-1, pdhb-1, dlat-1, acly-1, acly-2
Tricarboxylic acid metabolic process	31.05	3.20E-10	aco-1, idhg-1, idh-1, cts-1, acly-1, acly-2

GO term enrichment from top 500 mass spectrometry proteins (Negative control)			
GO term	Fold Enrichment	p-value	Representative associated genes
L-amino acid catabolic process	13.85	1.27E-14	dlst-1, got-2.1, ahcy-1, mccc-1, gdh-1
Proteinogenic amino acid catabolic process	12.85	4.86E-14	dlst-1, got-2.1, got-2.2, F02A9.10, mccc-1
Alanine catabolic process	44.16	5.12E-04	C32F10.8
L-alanine metabolic process	44.16	5.12E-04	C32F10.8
Regulation of protein localization to the ER	44.16	5.12E-04	icd-1, icd-2
Negative regulation of protein localization	11.04	1.24E-05	icd-1, icd-2, cdc-48.2, ftt-2
Ketone body metabolic process	29.44	1.51E-03	hphd-1, C05C10.3
Fatty acid derived catabolic process	29.44	1.51E-03	hphd-1, C05C10.3
Anatomical structure morphogenesis	5.52	2.43E-07	myo-3, unc-54, act-5, erm-1, unc-15, emb-8
Tube development	3.68	1.51E-03	lam-1, hda-1, sma-1, let-418, pyr-1, spt-16
Purine ribonucleoside triphosphate biosynthetic process	14.13	5.02E-08	asb-1, atp-1, T22D1.3, Y69A2AR.18, atp-2
Purine ribonucleotide biosynthetic process	7.18	2.75E-08	gmps-1, atp-1, T22D1.3, Y69A2AR.18, adsl-1,adss-1
GTP metabolic process	29.44	1.51E-03	T22D1.3, ndk-1
AMP biosynthetic process	26.49	1.12E-04	adsl-1, adss-1, adk-1
Mitotic spindle organization	7.68	7.97E-06	spd-5, paa-1, tpxl-1, let-92, cdc-48.2, dhc-1

B

Enrichment of centromere-related genes	
Gene	Enrichment Ratio
nefr-1	121.41637
hcp-3	13.26415418
ska-3	26.52830837
san-1	7.937375803
ndc-80	6.581222193
mis-12	6.452589214
ska-1	5.732842823
mdf-1	4.376921342
rod-1	3.577183895
knl-2	3.080405495
fzy-1	2.955127307
cls-2	2.845736138
plk-1	2.81279933
kbp-5	2.679961418
klp-7	2.495102451
hcp-6	2.349041227
czw-1	2.335227449
kbp-1	1.983611933
smc-4	1.895932272
hcp-4	1.727331268
him-10	1.713225622
bub-1	1.694628825
mdf-2	1.686378382
mel-28	1.641716674
lis-1	1.504390021
kbp-2	1.489298728
mix-1	1.361745056
knl-1	1.242814502
hcp-1	1.17379193
xwl-1	1.156735494
hcp-2	1.136039254
kbp-4	1.060791731
bub-3	1.016521348
knl-3	0.953266723
kbp-3	0.880508413

C

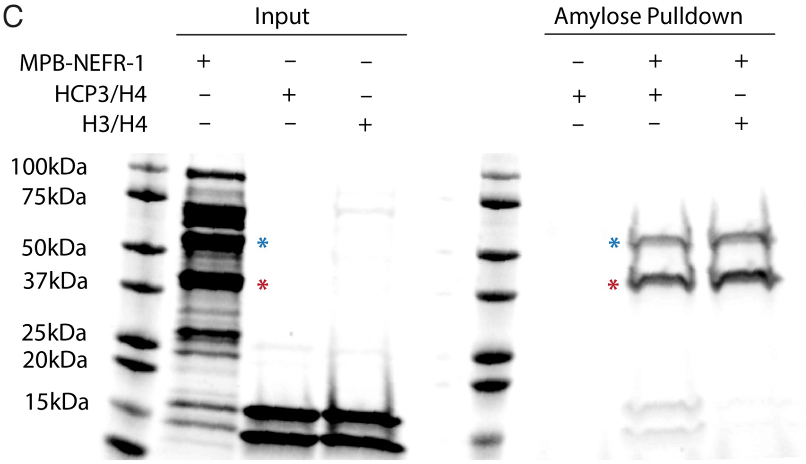

Figure S10. NEFR-1 physically associates with CENPA^{HCP-3}. A. Top 15 gene ontology (GO) analysis terms of mass spectrometry peptides identified in anti-FLAG immunoprecipitations from worm embryo lysate of either *3xFLAG::nefr-1* (left) or untagged control (right) strains. Tagged strains show specific enrichment of kinetochore and cell cycle-related genes. **B.** Normalized sum PEP score enrichment ratio of centromere-related genes[63] in *3xFLAG::nefr-1* immunoprecipitations over untagged controls. Genes highlighted in green, including CENPA^{HCP-3} and NEFR-1, were identified exclusively in *3xFLAG::nefr-1* immunoprecipitations. **C.** Amylose pulldown assay with purified MBP-NEFR-1 and HCP-3/H4 or canonical H3/H4 tetramers. MBP-NEFR-1 interacts directly with HCP-3/H4 tetramers, as suggested by co-precipitation of species at HCP-3 and H4 sizes. Blue asterisk indicates MBP-NEFR-1; red asterisk indicates a non-NEFR-1 contaminant.

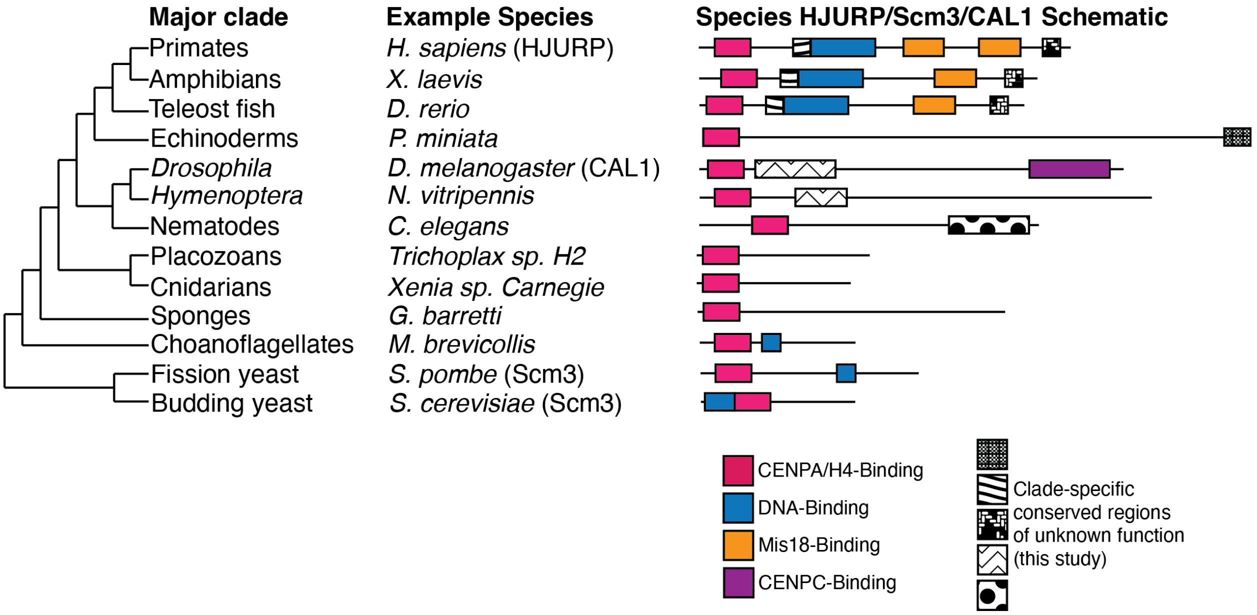

Figure S11. scm3 domain-containing proteins are present in most metazoan clades. We identify candidate HJURP orthologs across the metazoan tree and describe conserved domains previously unreported in these proteins that have yet to be functionally characterized.

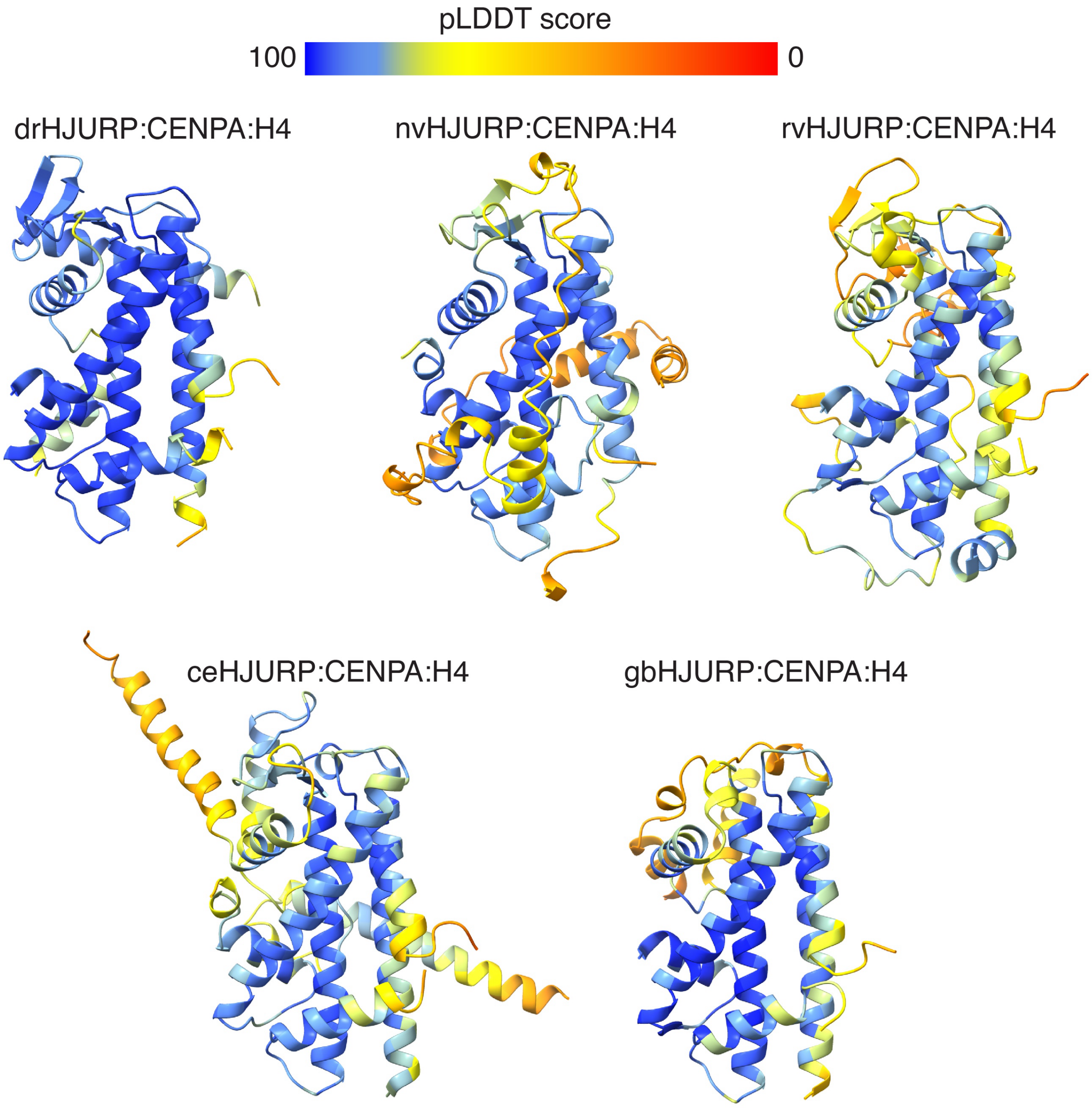

Figure S12. AlphaFold predictions colored by pLDDT score. All AlphaFold predictions presented in this study showed moderate to high confidence as measured by pLDDT (>80 at helical regions).

Table S1. A representative list of scm3 domain-containing proteins across the opisthokont lineage.

Table S2. Nucleic acid constructs used for this study.

Table S3. A list of *C. elegans* strains generated and/or used in this study.

Table S4. Sum PEP score ratios from 3xFLAG:NEFR-1 IP-MS data compared to the untagged control.

Data S1. Hidden Markov model (HMM) alignments used for this study.

Data S2. Protein alignments used for the generation of logo plots and trees presented in this study.

Data S3. Codon alignments used for the analyses in Figure 6E.

Movie S1. Timelapse of *D. rerio* embryo expressing GFP-CENPA and mCherry-HJURP shows discrete, cell cycle-dependent colocalization.

REFERENCES

1. K. L. McKinley, I. M. Cheeseman, The molecular basis for centromere identity and function. *Nat. Rev. Mol. Cell Biol.* **17**, 16–29 (2016).

2. L. Li, Q. Yuan, Y.-M. Chu, H.-Y. Jiang, J.-H. Zhao, Q. Su, D.-Q. Huo, X.-F. Zhang, Advances in holliday junction recognition protein (HJURP): Structure, molecular functions, and roles in cancer. *Front. Cell Dev. Biol.* **11**, 1106638 (2023).

3. S. Stoler, K. Rogers, S. Weitze, L. Morey, M. Fitzgerald-Hayes, R. E. Baker, Scm3, an essential *Saccharomyces cerevisiae* centromere protein required for G2/M progression and Cse4 localization. *Proc. Natl. Acad. Sci. U.S.A.* **104**, 10571–10576 (2007).

4. R. Camahort, B. Li, L. Florens, S. K. Swanson, M. P. Washburn, J. L. Gerton, Scm3 is essential to recruit the histone h3 variant cse4 to centromeres and to maintain a functional kinetochore. *Mol. Cell* **26**, 853–865 (2007).

5. G. Mizuguchi, H. Xiao, J. Wisniewski, M. M. Smith, C. Wu, Nonhistone Scm3 and histones CenH3-H4 assemble the core of centromere-specific nucleosomes. *Cell* **129**, 1153–1164 (2007).

6. D. R. Foltz, L. E. T. Jansen, A. O. Bailey, J. R. Yates III, E. A. Bassett, S. Wood, B. E. Black, D. W. Cleveland, Centromere-specific assembly of CENP-a nucleosomes is mediated by HJURP. *Cell* **137**, 472–484 (2009).

7. E. M. Dunleavy, D. Roche, H. Tagami, N. Lacoste, D. Ray-Gallet, Y. Nakamura, Y. Daigo, Y. Nakatani, G. Almouzni-Pettinotti, HJURP is a cell-cycle-dependent maintenance and deposition factor of CENP-A at centromeres. *Cell* **137**, 485–497 (2009).

8. M. C. Barnhart, P. H. J. L. Kuich, M. E. Stellfox, J. A. Ward, E. A. Bassett, B. E. Black, D. R. Foltz, HJURP is a CENP-A chromatin assembly factor sufficient to form a functional de novo kinetochore. *J. Cell Biol.* **194**, 229–243 (2011).

9. L. Sanchez-Pulido, A. L. Pidoux, C. P. Ponting, R. C. Allshire, Common ancestry of the CENP-A chaperones Scm3 and HJURP. *Cell* **137**, 1173–1174 (2009).

10. Z. Zhou, H. Feng, B.-R. Zhou, R. Ghirlando, K. Hu, A. Zwolak, L. M. Miller Jenkins, H. Xiao, N. Tjandra, C. Wu, Y. Bai, Structural basis for recognition of centromere histone variant CenH3 by the chaperone Scm3. *Nature* **472**, 234–237 (2011).

11. U.-S. Cho, S. C. Harrison, Recognition of the centromere-specific histone Cse4 by the chaperone Scm3. *Proc. Natl. Acad. Sci. U.S.A.* **108**, 9367–9371 (2011).

12. H. Hu, Y. Liu, M. Wang, J. Fang, H. Huang, N. Yang, Y. Li, J. Wang, X. Yao, Y. Shi, G. Li, R.-M. Xu, Structure of a CENP-A-histone H4 heterodimer in complex with chaperone HJURP. *Genes Dev.* **25**, 901–906 (2011).

13. M. Shuaib, K. Ouararhni, S. Dimitrov, A. Hamiche, HJURP binds CENP-A via a highly conserved N-terminal domain and mediates its deposition at centromeres. *Proc. Natl. Acad. Sci. U.S.A.* **107**, 1349–1354 (2010).

14. A. R. Popchock, S. Hedouin, Y. Mao, C. L. Asbury, A. B. Stergachis, S. Biggins, Stable centromere association of the yeast histone variant Cse4 requires its essential N-terminal domain. *EMBO J.* **44**, 1488–1511 (2025).

15. P. Agarwal, A. Alekar, S. Mallick, J. Shah, M. Basrai, S. K. Ghosh, Scm3 interacts with the N-terminal tail of Cse4 to regulate kinetochore assembly in budding yeast, bioRxiv [Preprint] (2025). https://doi.org/10.1101/2025.10.20.683541.

16. S. Le Goff, B. N. Keçeli, H. Jeřábková, S. Heckmann, T. Rutten, S. Cotterell, V. Schubert, E. Roitinger, K. Mechtler, F. C. H. Franklin, C. Tatout, A. Houben, D. Geelen, A. V. Probst, I. Lermontova, The H3 histone chaperone NASPSIM3 escorts CenH3 in *Arabidopsis*. *Plant J.* **101**, 71–86 (2020).

17. H. Takeuchi, S. Nagahara, T. Higashiyama, F. Berger, The chaperone NASP contributes to de Novo deposition of the centromeric histone variant CENH3 in *Arabidopsis* early embryogenesis. *Plant Cell Physiol.* **65**, 1135–1148 (2024).

18. B. E. Black, D. R. Foltz, S. Chakravarthy, K. Luger, V. L. Woods Jr., D. W. Cleveland, Structural determinants for generating centromeric chromatin. *Nature* **430**, 578–582 (2004).

19. B. E. Black, L. E. T. Jansen, P. S. Maddox, D. R. Foltz, A. B. Desai, J. V. Shah, D. W. Cleveland, Centromere identity maintained by nucleosomes assembled with histone H3 containing the CENP-A targeting domain. *Mol. Cell* **25**, 309–322 (2007).

20. K. L. McKinley, I. M. Cheeseman, Polo-like kinase 1 licenses CENP-A deposition at centromeres. *Cell* **158**, 397–411 (2014).

21. Y. Fujita, T. Hayashi, T. Kiyomitsu, Y. Toyoda, A. Kokubu, C. Obuse, M. Yanagida, Priming of centromere for CENP-A recruitment by human hMis18alpha, hMis18beta, and M18BP1. *Dev. Cell* **12**, 17–30 (2007).

22. D. Pan, K. Walstein, A. Take, D. Bier, N. Kaiser, A. Musacchio, Mechanism of centromere recruitment of the CENP-A chaperone HJURP and its implications for centromere licensing. *Nat. Commun.* **10**, 1–18 (2019).

23. T. Hori, Y. Mahana, M. Ariyoshi, T. Fukagawa, Dual pathways via CENP-C and Mis18C recruit HJURP for CENP-A deposition into vertebrate centromeres. *EMBO J.* **45**, 1292–1331 (2026).

24. H. Tachiwana, S. Müller, J. Blümer, K. Klare, A. Musacchio, G. Almouzni, HJURP involvement in de novo CenH3 (CENP-A) and CENP-C recruitment. *Cell Rep.* **11**, 22–32 (2015).

25. C. de Groot, J. Houston, B. Davis, A. Gerson-Gurwitz, J. Monen, P. Lara-Gonzalez, K. Oegema, A. K. Shiau, A. Desai, The N-terminal tail of *C. elegans* CENP-A interacts with KNL-2 and is essential for centromeric chromatin assembly. *Mol. Biol. Cell* **32**, 1193–1201 (2021).

26. P. S. Maddox, F. Hyndman, J. Monen, K. Oegema, A. Desai, Functional genomics identifies a Myb domain-containing protein family required for assembly of CENP-A chromatin. *J. Cell Biol.* **176**, 757–763 (2007).

27. R. F. Prosée, J. M. Wenda, I. Özdemir, C. Gabus, K. Delaney, F. Schwager, M. Gotta, F. A. Steiner, Transgenerational inheritance of centromere identity requires the CENP-A N-terminal tail in the *C. elegans* maternal germ line. *PLOS Biol.* **19**, e3000968 (2021).

28. R. S. Keegan, E. M. Dunleavy, Distinct pools of CENP-A and CENP-C support unique phases of centromere assembly in spermatogenesis, bioRxiv [Preprint] (2026) p. 2026.01.07.698108.

29. L. F. Rosin, B. G. Mellone, Centromeres drive a hard bargain. *Trends Genet.* **33**, 101–117 (2017).

30. H. S. Malik, S. Henikoff, Phylogenomics of the nucleosome. *Nat. Struct. Biol.* **10**, 882–891 (2003).

31. R. Phansalkar, P. Lapierre, B. G. Mellone, Evolutionary insights into the role of the essential centromere protein CAL1 in *Drosophila*. *Chromosome Res.* **20**, 493–504 (2012).

32. S. Erhardt, B. G. Mellone, C. M. Betts, W. Zhang, G. H. Karpen, A. F. Straight, Genome-wide analysis reveals a cell cycle-dependent mechanism controlling centromere propagation. *J. Cell Biol.* **183**, 805–818 (2008).

33. B. G. Mellone, K. J. Grive, V. Shteyn, S. R. Bowers, I. Oderberg, G. H. Karpen, Assembly of *Drosophila* centromeric chromatin proteins during mitosis. *PLOS Genet.* **7**, e1002068 (2011).

34. C.-C. Chen, M. L. Dechassa, E. Bettini, M. B. Ledoux, C. Belisario, P. Heun, K. Luger, B. G. Mellone, CAL1 is the *Drosophila* CENP-A assembly factor. *J. Cell Biol.* **204**, 313–329 (2014).

35. L. E. Kursel, F. C. Welsh, H. S. Malik, Ancient coretention of paralogs of Cid centromeric histones and Cal1 chaperones in mosquito species. *Mol. Biol. Evol.* **37**, 1949–1963 (2020).

36. B. Medina-Pritchard, V. Lazou, J. Zou, O. Byron, M. A. Abad, J. Rappsilber, P. Heun, A. A. Jeyaprakash, Structural basis for centromere maintenance by *Drosophila* CENP-A chaperone CAL1. *EMBO J.* **39**, e103234 (2020).

37. Z. Lin, K. W. Y. Yuen, RbAp46/48LIN-53 and HAT-1 are required for initial CENP-AHCP-3 deposition and de novo holocentromere formation on artificial chromosomes in *Caenorhabditis elegans* embryos. *Nucleic Acids Res.* **49**, 9154–9173 (2021).

38. J. Chen, M. Guo, X. Wang, B. Liu, A comprehensive review and comparison of different computational methods for protein remote homology detection. *Brief. Bioinform.* **19**, 231–244 (2018).

39. P. Radivojac, Advancing remote homology detection: A step toward understanding and accurately predicting protein function. *Cell Syst.* **13**, 435–437 (2022).

40. J. Abramson, J. Adler, J. Dunger, R. Evans, T. Green, A. Pritzel, O. Ronneberger, L. Willmore, A. J. Ballard, J. Bambrick, S. W. Bodenstein, D. A. Evans, C.-C. Hung, M. O'Neill, D. Reiman, K. Tunyasuvunakool, Z. Wu, A. Žemgulytė, E. Arvaniti, C. Beattie, O. Bertolli, A. Bridgland, A. Cherepanov, M. Congreve, A. I. Cowen-Rivers, A. Cowie, M. Figurnov, F. B. Fuchs, H. Gladman, R. Jain, Y. A. Khan, C. M. R. Low, K. Perlin, A. Potapenko, P. Savy, S. Singh, A. Stecula, A. Thillaisundaram, C. Tong, S. Yakneen, E. D. Zhong, M. Zielinski, A. Žídek, V. Bapst, P. Kohli, M. Jaderberg, D. Hassabis, J. M. Jumper, Accurate structure prediction of biomolecular interactions with AlphaFold 3. *Nature* **630**, 493–500 (2024).

41. R. Evans, M. O'Neill, A. Pritzel, N. Antropova, A. Senior, T. Green, A. Žídek, R. Bates, S. Blackwell, J. Yim, O. Ronneberger, S. Bodenstein, M. Zielinski, A. Bridgland, A. Potapenko, A. Cowie, K. Tunyasuvunakool, R. Jain, E. Clancy, P. Kohli, J. Jumper, D. Hassabis, Protein complex prediction with AlphaFold-Multimer, bioRxiv [Preprint] (2022) p. 2021.10.04.463034.

42. S. Hotaling, J. L. Kelley, P. B. Frandsen, Toward a genome sequence for every animal: Where are we now? *Proc. Natl. Acad. Sci. U.S.A.* **118**, e2109019118 (2021).

43. S. F. Altschul, T. L. Madden, A. A. Schäffer, J. Zhang, Z. Zhang, W. Miller, D. J. Lipman, Gapped BLAST and PSI-BLAST: A new generation of protein database search programs. *Nucleic Acids Res.* **25**, 3389–3402 (1997).

44. C. T. Amemiya, J. Alföldi, A. P. Lee, S. Fan, H. Philippe, I. MacCallum, I. Braasch, T. Manousaki, I. Schneider, N. Rohner, C. Organ, D. Chalopin, J. J. Smith, M. Robinson, R. A. Dorrington, M. Gerdol, B. Aken, M. A. Biscotti, M. Barucca, D. Baurain, A. M. Berlin, G. L. Blatch, F. Buonocore, T. Burmester, M. S. Campbell, A. Canapa, J. P. Cannon, A. Christoffels, G. De Moro, A. L. Edkins, L. Fan, A. M. Fausto, N. Feiner, M. Forconi, J. Gamieldien, S. Gnerre, A. Gnirke, J. V. Goldstone, W. Haerty, M. E. Hahn, U. Hesse, S. Hoffmann, J. Johnson, S. I. Karchner, S. Kuraku, M. Lara, J. Z. Levin, G. W. Litman, E. Mauceli, T. Miyake, M. G. Mueller, D. R. Nelson, A. Nitsche, E. Olmo, T. Ota, A. Pallavicini, S. Panji, B. Picone, C. P. Ponting, S. J. Prohaska, D. Przybylski, N. R. Saha, V. Ravi, F. J. Ribeiro, T. Sauka-Spengler, G. Scapigliati, S. M. J. Searle, T. Sharpe, O. Simakov, P. F. Stadler, J. J. Stegeman, K. Sumiyama, D. Tabbaa, H. Tafer, J. Turner-Maier, P. van Heusden, S. White, L. Williams, M. Yandell, H. Brinkmann, J.-N. Volff, C. J. Tabin, N. Shubin, M. Schartl, D. B. Jaffe, B. Venkatesh, F. Di Palma, E. S. Lander, A. Meyer, K. Lindblad-Toh, The African coelacanth genome provides insights into tetrapod evolution. *Nature* **496**, 311–316 (2013).

45. R. C. Edgar, MUSCLE: A multiple sequence alignment method with reduced time and space complexity. *BMC Bioinformatics* **5**, 113 (2004).

46. M. Blum, A. Andreeva, L. C. Florentino, S. R. Chuguransky, T. Grego, E. Hobbs, B. L. Pinto, A. Orr, T. Paysan-Lafosse, I. Ponamareva, G. A. Salazar, N. Bordin, P. Bork, A. Bridge, L. Colwell, J. Gough, D. H. Haft, I. Letunic, F. Llinares-López, A. Marchler-Bauer, L. Meng-Papaxanthos, H. Mi, D. A. Natale, C. A. Orengo, A. P. Pandurangan, D. Piovesan, C. Rivoire, C. J. A. Sigrist, N. Thanki, F. Thibaud-Nissen, P. D. Thomas, S. C. E. Tosatto, C. H. Wu, A. Bateman, InterPro: The protein sequence classification resource in 2025. *Nucleic Acids Res.* **53**, D444–D456 (2025).

47. B. J. Swalla, Deuterostome ancestors and chordate origins. *Integr. Comp. Biol.* **64**, 1175–1181 (2024).

48. H. Luo, F. Gao, Y. Lin, Evolutionary conservation analysis between the essential and nonessential genes in bacterial genomes. *Sci. Rep.* **5**, 13210 (2015).

49. J. Söding, A. Biegert, A. N. Lupas, The HHpred interactive server for protein homology detection and structure prediction. *Nucleic Acids Res.* **33**, W244–W248 (2005).

50. R. Chikhi, T. Lemane, R. Loll-Krippleber, M. Montoliu-Nerin, B. Raffestin, A. P. Camargo, C. J. Miller, M. B. Fiamenghi, D. P. Agustinho, S. Majidian, G. Autric, M. Hugues, J. Lee, R. Faure, K. D. Curry, J. A. Moura de Sousa, E. P. C. Rocha, D. Koslicki, P. Medvedev, P. Gupta, J. Shen, A. Morales-Tapia, K. Sihuta, P. J. Roy, G. W. Brown, R. C. Edgar, A. Korobeynikov, M. Steinegger, C. A. Lareau, P. Peterlongo, A. Babaian, Logan: Planetary-scale genome assembly surveys life's diversity*, bioRxivorg* (2025) p. 2024.07.30.605881.

51. A. Pallavicini, A. Canapa, M. Barucca, J. Alfőldi, M. A. Biscotti, F. Buonocore, G. De Moro, F. Di Palma, A. M. Fausto, M. Forconi, M. Gerdol, D. M. Makapedua, J. Turner-Meier, E. Olmo, G. Scapigliati, Analysis of the transcriptome of the Indonesian coelacanth *Latimeria menadoensis*. *BMC Genomics* **14**, 538 (2013).

52. J. Sprague, D. Clements, T. Conlin, P. Edwards, K. Frazer, K. Schaper, E. Segerdell, P. Song, B. Sprunger, M. Westerfield, The Zebrafish Information Network (ZFIN): The zebrafish model organism database. *Nucleic Acids Res.* **31**, 241–243 (2003).

53. E. A. Bassett, J. DeNizio, M. C. Barnhart-Dailey, T. Panchenko, N. Sekulic, D. J. Rogers, D. R. Foltz, B. E. Black, HJURP uses distinct CENP-A surfaces to recognize and to stabilize CENP-A/histone H4 for centromere assembly. *Dev. Cell* **22**, 749–762 (2012).

54. T. Hori, J. Cao, K. Nishimura, M. Ariyoshi, Y. Arimura, H. Kurumizaka, T. Fukagawa, Essentiality of CENP-A depends on its binding mode to HJURP. *Cell Rep.* **33**, 108388 (2020).

55. R. Bernad, P. Sánchez, T. Rivera, M. Rodríguez-Corsino, E. Boyarchuk, I. Vassias, D. Ray-Gallet, A. Arnaoutov, M. Dasso, G. Almouzni, A. Losada, Xenopus HJURP and condensin II are required for CENP-A assembly. *J. Cell Biol.* **192**, 569–582 (2011).

56. M. Bui, E. K. Dimitriadis, C. Hoischen, E. An, D. Quénet, S. Giebe, A. Nita-Lazar, S. Diekmann, Y. Dalal, Cell-cycle-dependent structural transitions in the human CENP-A nucleosome in vivo. *Cell* **150**, 317–326 (2012).

57. A. Sur, Y. Wang, P. Capar, G. Margolin, M. K. Prochaska, J. A. Farrell, Single-cell analysis of shared signatures and transcriptional diversity during zebrafish development. *Dev. Cell* **58**, 3028–3047.e12 (2023).

58. D. R. Farnsworth, L. M. Saunders, A. C. Miller, A single-cell transcriptome atlas for zebrafish development. *Dev. Biol.* **459**, 100–108 (2020).

59. B. Thisse, S. Pflumio, M. Fürthauer, B. Loppin, V. Heyer, A. Degrave, R. Woehl, A. Lux, T. Steffan, X. Q. Charbonnier, C. Thisse, "Expression of the zebrafish genome during embryogenesis" (R01RR15402, NIH).

60. R. B. Schittenhelm, F. Althoff, S. Heidmann, C. F. Lehner, Detrimental incorporation of excess Cenp-A/Cid and Cenp-C into *Drosophila* centromeres is prevented by limiting amounts of the bridging factor Cal1. *J. Cell Sci.* **123**, 3768–3779 (2010).

61. D. L. Bodor, L. P. Valente, J. F. Mata, B. E. Black, L. E. T. Jansen, Assembly in G1 phase and long-term stability are unique intrinsic features of CENP-A nucleosomes. *Mol. Biol. Cell* **24**, 923–932 (2013).

62. K. Karplus, C. Barrett, R. Hughey, Hidden Markov models for detecting remote protein homologies. *Bioinformatics* **14**, 846–856 (1998).

63. S. R. Eddy, "HMMER user's guide" (2023); http://eddylab.org/software/hmmer/Userguide.pdf.

64. S. C. Potter, A. Luciani, S. R. Eddy, Y. Park, R. Lopez, R. D. Finn, HMMER web server: 2018 update. *Nucleic Acids Res.* **46**, W200–W204 (2018).

65. L. Rosin, B. G. Mellone, Co-evolving CENP-A and CAL1 domains mediate centromeric CENP-A deposition across *Drosophila* species. *Dev. Cell* **37**, 136–147 (2016).

66. B. L. Carty, A. A. Dattoli, E. M. Dunleavy, CENP-C functions in centromere assembly, the maintenance of CENP-A asymmetry and epigenetic age in *Drosophila* germline stem cells. *PLOS Genet.* **17**, e1009247 (2021).

67. Y. Unhavaithaya, T. L. Orr-Weaver, Centromere proteins CENP-C and CAL1 functionally interact in meiosis for centromere clustering, pairing, and chromosome segregation. *Proc. Natl. Acad. Sci. U.S.A.* **110**, 19878–19883 (2013).

68. R. Kitagawa, Key players in chromosome segregation in *Caenorhabditis elegans*. *Front. Biosci. (Landmark Ed)* **14**, 1529–1557 (2009).

69. B. C. H. Lee, Z. Lin, K. W. Y. Yuen, RbAp46/48 (LIN-53) is required for holocentromere assembly in *Caenorhabditis elegans*. *Cell Rep.* **14**, 1819–1828 (2016).

70. S. Tandonnet, M. Haq, A. Turner, T. Grana, P. Paganopoulou, S. Adams, S. Dhawan, N. Kanzaki, I. Nuez, M.-A. Félix, A. Pires-daSilva, *De Novo* genome assembly of *Auanema melissensis*, a trioecious free-living nematode. *J. Nematol.* **54**, 20220059 (2022).

71. P. Bhan, M. Muthaiyan Shanmugam, D. Wang, O. Bayansan, C.-W. Chen, O. I. Wagner, Characterization of TAG-63 and its role on axonal transport in C. elegans. *Traffic* **21**, 231–249 (2020).

72. X. Qing, Y. M. Zhang, S. Sun, M. Ahmed, W.-S. Lo, W. Bert, O. Holovachov, H. Li, Phylogenomic insights into the evolution and origin of Nematoda. *Syst. Biol.* **74**, 349–358 (2025).

73. A. Ghaddar, E. Armingol, C. Huynh, L. Gevirtzman, N. E. Lewis, R. Waterston, E. J. O'Rourke, Whole-body gene expression atlas of an adult metazoan. *Sci. Adv.* **9**, eadg0506 (2023).

74. J. S. Packer, Q. Zhu, C. Huynh, P. Sivaramakrishnan, E. Preston, H. Dueck, D. Stefanik, K. Tan, C. Trapnell, J. Kim, R. H. Waterston, J. I. Murray, A lineage-resolved molecular atlas of *C. elegans* embryogenesis at single-cell resolution. *Science* **365**, eaax1971 (2019).

75. T. Gong, F. J. McNally, *Caenorhabditis elegans* spermatocytes can segregate achiasmate homologous chromosomes apart at higher than random frequency during meiosis I. *Genetics* **223**, iyad021 (2023).

76. K. Oegema, A. Desai, S. Rybina, M. Kirkham, A. A. Hyman, Functional analysis of kinetochore assembly in *Caenorhabditis elegans*. *J. Cell Biol.* **153**, 1209–1226 (2001).

77. B. J. Buchwitz, K. Ahmad, L. L. Moore, M. B. Roth, S. Henikoff, A histone-H3-like protein in *C. elegans*: Cell division. *Nature* **401**, 547–548 (1999).

78. G. E. Ashley, T. Duong, M. T. Levenson, M. A. Q. Martinez, L. C. Johnson, J. D. Hibshman, H. N. Saeger, N. J. Palmisano, R. Doonan, R. Martinez-Mendez, B. R. Davidson, W. Zhang, J. M. Ragle, T. N. Medwig-Kinney, S. S. Sirota, B. Goldstein, D. Q. Matus, D. J. Dickinson, D. J. Reiner, J. D. Ward, An expanded auxin-inducible degron toolkit for *Caenorhabditis elegans*. *Genetics* **217**, iyab006 (2021).

79. L. Zhang, J. D. Ward, Z. Cheng, A. F. Dernburg, The auxin-inducible degradation (AID) system enables versatile conditional protein depletion in *C. elegans*. *Development* **142**, 4374–4384 (2015).

80. R. Gassmann, A. Rechtsteiner, K. W. Yuen, A. Muroyama, T. Egelhofer, L. Gaydos, F. Barron, P. Maddox, A. Essex, J. Monen, S. Ercan, J. D. Lieb, K. Oegema, S. Strome, A. Desai, An inverse relationship to germline transcription defines centromeric chromatin in *C. elegans*. *Nature* **484**, 534–537 (2012).

81. E. M. Dunleavy, N. L. Beier, W. Gorgescu, J. Tang, S. V. Costes, G. H. Karpen, The cell cycle timing of centromeric chromatin assembly in *Drosophila meiosis* is distinct from mitosis yet requires CAL1 and CENP-C. *PLoS Biol.* **10**, e1001460 (2012).

82. S. J. Mondo, I. V. Grigoriev, A genomic perspective on fungal diversity and evolution. *Nat. Rev. Microbiol.* **23**, 718–733 (2025).

83. M. Carr, B. S. C. Leadbeater, R. Hassan, M. Nelson, S. L. Baldauf, Molecular phylogeny of choanoflagellates, the sister group to Metazoa. *Proc. Natl. Acad. Sci. U.S.A.* **105**, 16641–16646 (2008).

84. C. W. Dunn, G. Giribet, G. D. Edgecombe, A. Hejnol, Animal phylogeny and its evolutionary implications. *Annu. Rev. Ecol. Evol. Syst.* **45**, 371–395 (2014).

85. S. Henikoff, K. Ahmad, H. S. Malik, The centromere paradox: Stable inheritance with rapidly evolving DNA. *Science* **293**, 1098–1102 (2001).

86. H. S. Malik, S. Henikoff, Adaptive evolution of Cid, a centromere-specific histone in *Drosophila*. *Genetics* **157**, 1293–1298 (2001).

87. L. Piovani, D. Gavriouchkina, E. Parey, L. A. Sarre, K. T. C. A. Peijnenburg, J. M. Martín-Durán, D. S. Rokhsar, N. Satoh, A. de Mendoza, T. Goto, F. Marlétaz, The genomic origin of the unique chaetognath body plan. *Nature* **645**, 964–973 (2025).

88. I. A. Drinnenberg, D. deYoung, S. Henikoff, H. S. Malik, Recurrent loss of CenH3 is associated with independent transitions to holocentricity in insects. *eLife* **3**, e03676 (2014).

89. M. I. Navarro-Mendoza, C. Pérez-Arques, S. Panchal, F. E. Nicolás, S. J. Mondo, P. Ganguly, J. Pangilinan, I. V. Grigoriev, J. Heitman, K. Sanyal, V. Garre, Early diverging fungus Mucor circinelloides lacks centromeric histone CENP-A and displays a mosaic of point and regional centromeres. *Curr. Biol.* **29**, 3791–3802.e6 (2019).

90. T. Kumon, J. Ma, R. B. Akins, D. Stefanik, C. E. Nordgren, J. Kim, M. T. Levine, M. A. Lampson, Parallel pathways for recruiting effector proteins determine centromere drive and suppression. *Cell* **184**, 4904–4918.e11 (2021).

91. C. M. Hammond, C. B. Strømme, H. Huang, D. J. Patel, A. Groth, Histone chaperone networks shaping chromatin function. *Nat. Rev. Mol. Cell Biol.* **18**, 141–158 (2017).

92. L. Sanchez-Pulido, C. P. Ponting, Extending the horizon of homology detection with coevolution-based structure prediction. *J. Mol. Biol.* **433**, 167106 (2021).

93. C. M. Weisman, A. W. Murray, S. R. Eddy, Many, but not all, lineage-specific genes can be explained by homology detection failure. *PLoS Biol.* **18**, e3000862 (2020).

94. C. Yu, S. R. Sankaranarayanan, G. Cornilleau, A. C. Howes, C. Azumaya, I. Zilberleyb, B. Brillantes, T. K. Cheung, L. Dec, D. Loew, P. Tran, C. M. Rose, I. A. Drinnenberg, C. Ciferri, S. Yatskevich, Architecture and function of holocentric CENP-A-independent kinetochores, bioRxiv [Preprint] (2025)p. 2025.07.14.664805.

95. S. Lidgard, A. C. Love, Rethinking living fossils. *Bioscience* **68**, 760–770 (2018).

96. M. Perpelescu, T. Hori, A. Toyoda, S. Misu, N. Monma, K. Ikeo, C. Obuse, A. Fujiyama, T. Fukagawa, HJURP is involved in the expansion of centromeric chromatin. *Mol. Biol. Cell* **26**, 2742–2754 (2015).

97. S. Müller, R. Montes de Oca, N. Lacoste, F. Dingli, D. Loew, G. Almouzni, Phosphorylation and DNA binding of HJURP determine its centromeric recruitment and function in CenH3 (CENP-A) loading. *Cell Rep.* **8**, 190–203 (2014).

98. H. Xiao, G. Mizuguchi, J. Wisniewski, Y. Huang, D. Wei, C. Wu, Nonhistone Scm3 binds to AT-rich DNA to organize atypical centromeric nucleosome of budding yeast. *Mol. Cell* **43**, 369–380 (2011).

99. P. Agarwal, A. Alekar, S. Mallick, K. Harini, S. K. Ghosh, Evidence of centromeric histone 3 chaperone involved in DNA damage repair pathway in budding yeast. *eLife* **14**, e104431 (2025).

100. H. D. Folco, H. Xiao, D. Wheeler, H. Feng, Y. Bai, S. I. S. Grewal, The cysteine-rich domain in CENP-A chaperone Scm3HJURP ensures centromere targeting and kinetochore integrity. *Nucleic Acids Res.* **52**, 1688–1701 (2024).

101. B. T. French, F. G. Westhorpe, C. Limouse, A. F. Straight, Xenopus laevis M18BP1 directly binds existing CENP-A nucleosomes to promote centromeric chromatin assembly. *Dev. Cell* **42**, 190–199.e10 (2017).

102. M. A. Lampson, B. E. Black, Cellular and molecular mechanisms of centromere drive. *Cold Spring Harb. Symp. Quant. Biol.* **82**, 249–257 (2017).

103. T. Kato, N. Sato, S. Hayama, T. Yamabuki, T. Ito, M. Miyamoto, S. Kondo, Y. Nakamura, Y. Daigo, Activation of Holliday junction recognizing protein involved in the chromosomal stability and immortality of cancer cells. *Cancer Res.* **67**, 8544–8553 (2007).

104. J. Helsen, K. Ramachandran, G. Sherlock, G. Dey, Progressive coevolution of the yeast centromere and kinetochore. *Nature* **651**, 1012–1019 (2026).

105. L. E. Kursel, H. S. Malik, The cellular mechanisms and consequences of centromere drive. *Curr. Opin. Cell Biol.* **52**, 58–65 (2018).

106. N. Chen, T. W. Harris, I. Antoshechkin, C. Bastiani, T. Bieri, D. Blasiar, K. Bradnam, P. Canaran, J. Chan, C.-K. Chen, W. J. Chen, F. Cunningham, P. Davis, E. Kenny, R. Kishore, D. Lawson, R. Lee, H.-M. Muller, C. Nakamura, S. Pai, P. Ozersky, A. Petcherski, A. Rogers, A. Sabo, E. M. Schwarz, K. Van Auken, Q. Wang, R. Durbin, J. Spieth, P. W. Sternberg, L. D. Stein, WormBase: A comprehensive data resource for *Caenorhabditis* biology and genomics. *Nucleic Acids Res.* **33**, D383–D389 (2005).

107. Z. Wang, H. Chen, S. Jin, Z. Li, Y. Mei, Y. Xiao, C. Zhao, T. Zhou, F. Li, Y. Liu, K. He, InsectBase 3.0: A comprehensive multi-omics resource for insects. *Nucleic Acids Res.* **54**, D1143–D1151 (2026).

108. E. F. Pettersen, T. D. Goddard, C. C. Huang, E. C. Meng, G. S. Couch, T. I. Croll, J. H. Morris, T. E. Ferrin, UCSF ChimeraX: Structure visualization for researchers, educators, and developers. *Protein Sci.* **30**, 70–82 (2021).

109. T. D. Schneider, R. M. Stephens, Sequence logos: A new way to display consensus sequences. *Nucleic Acids Res.* **18**, 6097–6100 (1990).

110. A. Löytynoja, N. Goldman, webPRANK: A phylogeny-aware multiple sequence aligner with interactive alignment browser. *BMC Bioinformatics* **11**, 579 (2010).

111. J. Trifinopoulos, L.-T. Nguyen, A. von Haeseler, B. Q. Minh, W-IQ-TREE: A fast online phylogenetic tool for maximum likelihood analysis. *Nucleic Acids Res.* **44**, W232–W235 (2016).

112. B. Q. Minh, H. A. Schmidt, O. Chernomor, D. Schrempf, M. D. Woodhams, A. von Haeseler, R. Lanfear, IQ-TREE 2: New models and efficient methods for phylogenetic inference in the genomic era. *Mol. Biol. Evol.* **37**, 1530–1534 (2020).

113. Z. Yang, PAML 4: Phylogenetic analysis by maximum likelihood. *Mol. Biol. Evol.* **24**, 1586–1591 (2007).

114. P. Raman, H. Khan, J. M. Young, T. Tsukiyama, H. S. Malik, Dynamic evolution of EZHIP, an inhibitor of the Polycomb Repressive Complex 2 in mammals, bioRxivorg [Preprint] (2025) p. 2025.12.12.693809.

115. J. C. F. Servin, A. F. Straight, Repression of CENP-A assembly in metaphase requires HJURP phosphorylation and inhibition by M18BP1, bioRxiv [Preprint] (2021). https://doi.org/10.1101/2021.10.28.466278.

116. M. Westerfield, *The Zebrafish Book. A Guide for the Laboratory Use of Zebrafish (Danio Rerio)* (Univ. of Oregon Press, 2000).

117. K. R. Siegfried, In search of determinants: Gene expression during gonadal sex differentiation. *J. Fish Biol.* **76**, 1879–1902 (2010).

118. R. S. Wu, I. I. Lam, H. Clay, D. N. Duong, R. C. Deo, S. R. Coughlin, A rapid method for directed gene knockout for screening in G0 zebrafish. *Dev. Cell* **46**, 112–125.e4 (2018).

119. J. C. Talbot, S. L. Amacher, A streamlined CRISPR pipeline to reliably generate zebrafish frameshifting alleles. *Zebrafish* **11**, 583–585 (2014).

120. J. N. Rosen, M. F. Sweeney, J. D. Mably, Microinjection of zebrafish embryos to analyze gene function. *J. Vis. Exp.* , 1115 (2009).

121. J. Schindelin, I. Arganda-Carreras, E. Frise, V. Kaynig, M. Longair, T. Pietzsch, S. Preibisch, C. Rueden, S. Saalfeld, B. Schmid, J.-Y. Tinevez, D. J. White, V. Hartenstein, K. Eliceiri, P. Tomancak, A. Cardona, Fiji: An open-source platform for biological-image analysis. *Nat. Methods* **9**, 676–682 (2012).

122. A. Paix, A. Folkmann, G. Seydoux, Precision genome editing using CRISPR-Cas9 and linear repair templates in C. elegans. *Methods* **121-122**, 86–93 (2017).

123. K. S. Ghanta, C. C. Mello, Melting dsDNA donor molecules greatly improves precision genome editing in *Caenorhabditis elegans*. *Genetics* **216**, 643–650 (2020).

124. A. E. Russo, S. Giacopazzi, A. Deshong, M. Menon, V. Ortiz, K. M. Ego, K. D. Corbett, N. Bhalla, The conserved AAA ATPase PCH-2 distributes its regulation of meiotic prophase events through multiple meiotic HORMADs in *C. elegans*. *PLOS Genet.* **19**, e1010708 (2023).

125. I. D. Wolff, N. S. Divekar, S. M. Wignall, Methods for investigating cell division mechanisms in *C. elegans*. *Methods Mol. Biol.* **2415**, 19–35 (2022).

126. L. Li, A. Y. Zinovyeva, Protein extract preparation and co-immunoprecipitation from *Caenorhabditis elegans*. *J. Vis. Exp.*, 10.3791/61243 (2020).

127. E. Zanin, J. Dumont, R. Gassmann, I. Cheeseman, P. Maddox, S. Bahmanyar, A. Carvalho, S. Niessen, J. R. Yates III, K. Oegema, A. Desai, Affinity purification of protein complexes in C. elegans. *Methods Cell Biol.* **106**, 289–322 (2011).

128. Lab, Multicellgenome (2019). Dudin et al. 2019. figshare. Dataset. https://doi.org/10.6084/m9.figshare.8299529.v2.

129. G. Torruella, A. de Mendoza, X. Grau-Bové, M. Antó, M. A. Chaplin, J. del Campo, L. Eme, G. Pérez-Cordón, C. M. Whipps, K. M. Nichols, R. Paley, A. J. Roger, A. Sitjà-Bobadilla, S. Donachie, I. Ruiz-Trillo, Phylogenomics reveals convergent evolution of lifestyles in close relatives of animals and fungi. *Curr. Biol.* **25**, 2404–2410 (2015).
